# Tyrosol effectively improves spermatogenesis recovery by S100A9/TLR4/NF-κB pathway in busulfan-induced oligozoospermia mice

**DOI:** 10.7717/peerj.20887

**Published:** 2026-03-30

**Authors:** Weizhen Wang, Wenbiao Zhou, Xiaoran Li, Taowen Ye, Wanqing Zhu, Guanning Zhong, Chengniu Wang, Haiying Geng, Xiaofang Tan, RuiKun Hu, Xiaorong Wang

**Affiliations:** 1 Center for Reproductive Medicine, Affiliated Maternity and Child Health Care Hospital of Nantong University, Nantong, Jiangsu, China; 2 Institute of Reproductive Medicine, Medical School, Nantong University, Nantong, Jiangsu, China; 3 Department of Clinical Laboratory, Affiliated Maternity and Child Health Care Hospital of Nantong University, Nantong, Jiangsu, China

**Keywords:** Tyrosol, Oligozoospermia, Male infertility, Anti-inflammatory, S100A9

## Abstract

**Background:**

Oligozoospermia is a prevalent clinical manifestation of male infertility. It has been provided to be bound up with testicular inflammation and oxidative stress. The phenolic compound 2-(4-hydroxyphenyl) ethanol, chemically designated as tyrosol (Tyr), has been widely distributed in multiple botanical sources, such as olive oil, *Rhodiola*, and other plants. Given its anti-inflammatory and antioxidant properties, Tyr shows potential as a pharmacological candidate for oligozoospermia. This research investigated the phenotype and molecular pathways associated with Tyr in mice.

**Methods:**

Adult mice were randomly assigned to six groups: a control group, a model group, a hydroxytyrosol (HT) group, and three Tyr groups (10 mg/kg, 30 mg/kg, and 50 mg/kg). All groups except the control group underwent a single does busulfan administration (30 mg/kg) *via* intraperitoneal injection to induce oligozoospermia. After a two-week observation period, the HT and Tyr groups were treated orally with their respective medications for four weeks. Blood, testes, epididymides, and sperm samples were then collected for further analysis.

**Results:**

Tyr effectively reversed the mice symptom of oligozoospermia, as demonstrated by increased testis weight, improved sperm concentration, and restored testicular histomorphology. In addition, Tyr normalized serum sex hormone levels and testicular oxidative indices. Moreover, it reduced testicular inflammation by decreasing IL-6 and TNF-α expression, likely through modulation of the S100A9/TLR4/NF-κB pathway.

**Conclusions:**

Tyr significantly ameliorated oligozoospermia in mice through S100A9/TLR4/NF-κB pathway, suggesting that it may represent a promising therapeutic option for patients with this condition.

## Introduction

Nowadays, infertility remains a significant global health issue, affecting 10–20% of couples of reproductive age in the world (World Health Organization, 2023). In approximately 30%–40% of these cases, male factors are the primary cause (Agarwal et al., 2015). Male infertility has complex causes, including nonobstructive causes (*e.g.*, testicular defects) and obstruction of the reproductive tract (The Practice Committee of the American Society for Reproductive Medicine, 2018; Modgil et al., 2016). Among these, abnormalities in sperm production and maturation are the most common, leading to poor semen quality, such as oligozoospermia, asthenozoospermia, teratozoospermia, azoospermia, or combinations thereof, including oligoteratozoospermia (Leslie, Soon-Sutton & Khan, 2024; Krausz, 2011).

Spermatogenesis is a complex process that depends on the balance of the testicular spermatogenic microenvironment, which includes a specialized immune microenvironment characterized by immune privilege and a local defense system (Zhao et al., 2014). The testicular immune microenvironment consists of spermatogonial, Sertoli, interstitial, peritubular myoid, and immune cells, which interact to form the immune environment of the testis (Meinhardt & Hedger, 2011). The blood-testis barrier (BTB), formed by Sertoli cells and peritubular myoid cells, acts as a barrier to isolate differentiated spermatogenic cells. It prevents sperm antigenicity from triggering an immune response and protects spermatogenesis from harmful substances (Zhao et al., 2014; Song et al., 2022). Androgens, secreted by Leydig cells, regulate the testicular immune-privileged state by modulating the functions of Sertoli cells and the expansion of T cells (Meng et al., 2011; Fijak et al., 2011). Furthermore, various immune cells are present in the testicular interstitium, with macrophages comprising 80% of the testicular intratesticular leukocytes, forming the first line of defense against microorganisms in the blood circulation (Indumathy et al., 2020).

There exist two types of macrophages in the testis: interstitial macrophages located in the testicular interstitium, and peritubular macrophages, found behind the basement membrane of the seminiferous tubule. Interstitial macrophages, also referred to M1-like macrophages due to their expression of anti-inflammatory cytokine genes such as IL10, are typically identified by CD206, CD64, CSF1R, along with reduced levels of MerTK (Gu et al., 2023; Lokka et al., 2020; Mossadegh-Keller et al., 2017; Wang et al., 2021). In contrast, peritubular macrophages are characterized by the absence or low levels of CD206, CD64, and CSF1R, as well as MerTK, and exhibit elevated levels of MHC II. These cells are classified as M1-like macrophages because of their significant transcriptional activation of pro-inflammatory mediators such as interleukin-6 (IL-6), tumor necrosis factor-α (TNF-α), and interleukin-1 β (IL-1β) (Gu et al., 2023; Lokka et al., 2020; Mossadegh-Keller et al., 2017; Wang et al., 2021).

Although the mammalian testis is a typical immune organ, the balance of its immune microenvironment can be disrupted under various pathological conditions, such as infection, testicular torsion, tissue damage, and tumors. This disruption may lead to autoimmune orchitis, which can impair spermatogenesis (Jacobo et al., 2011). Focal lymphocytic infiltrates are commonly observed in 25–30% of testicular biopsies associated with infertility (Schuppe et al., 2017). Clinically, the symptoms of autoimmune orchitis are often subtle, and no effective treatments are currently available (Silva et al., 2014). Therefore, identifying an effective therapeutic agent is crucial. Given that autoimmune orchitis frequently presents minimal symptoms and lacks effective treatments, the search for a therapeutic solution is of paramount importance. In recent years, natural products have emerged as potential treatments for abnormal spermatogenesis, with tyrosol, known for its anti-inflammatory properties, gaining attention.

Tyrosol (Tyr), 2-(4-hydroxyphenyl)-ethanol, a natural polyphenolic compound widely found in olive oil, rhodiola and other plants (St-Laurent-Thibault et al., 2011; Sun et al., 2012). It can reduce inflammatory and oxidative stress markers to improve the occurrence and development of many diseases. Several studies confirmed that tyrosol exerts beneficial effect in hypertension, hyperlipemia, hyperglycemia, atherosclerosis, coronary heart disease, acute cerebral ischemia-reperfusion, diabetes and obesity. For instance, in arterial inflammation and atherosclerosis, tyrosol are associated with reduction of lesion microcalcification (Zoubdane et al., 2024). In nonalcoholic fatty liver disease, experiments *in vivo* showed that Tyr contains the increase in hepatic lipid accumulation probably by the modulation of the PPARα signaling pathway (Wang et al., 2024b). In nephrotoxicity, Tyr effectively reduced creatinine and prevent nephrotoxicity due to antioxidant property (Zargari et al., 2023). In acute lung injury, Tyr attenuates the activation of inflammatory molecules and the expression of pro-inflammatory mediators for treating inflammatory lung diseases (Kim et al., 2017). These studies indicate that tyrosol can greatly improve inflammation, toxic damage and oxidative stress but its role in busulfan-induced oligozoospermia remains unknown.

A recent study demonstrated that Tyr activates the Nrf-2/HO-1 pathway to reverse AlCl_3_-induced testicular toxicity alterations (Güvenç et al., 2020). Here, we assumed that Tyr may improve busulfan-induced oligozoospermia in mice. To test this hypothesis, we employed various methods—including hematoxylin and eosin (HE) staining and computer-assisted sperm analysis (CASA)—to comprehensively evaluate the reproductive protective effects of Tyr in oligozoospermic mice and to determine its optimal dosage, using hydroxytyrosol as a positive control. Subsequently, enzyme-linked immunosorbent assay (ELISA) was utilized to assess the impact of Tyr on hormone levels and oxidative stress in oligozoospermic mice. Finally, high-throughput transcriptome sequencing and molecular experiments, including reverse transcription quantitative polymerase chain reaction (RT-qPCR), Western blot (WB) analysis and immunofluorescence (IF), were conducted to elucidate the potential mechanism of action of Tyr. This study extends our understanding of the pharmacological activities of tyrosol and may offer a novel candidate for reproductive protection.

## Materials and Methods

### Chemicals and reagents

Tyrosol (2-(4-hydroxyphenyl) ethanol) was acquired from Acmec Biochemical Technology (Shanghai, China) with a purity of > 97%. Busulfan (BUS) and dimethyl sulfoxide (DMSO) were bought through Sigma-Aldrich (Shanghai, China). The Periodic Acid-Schiff (PAS) stain kit was acquired from Solibio (Beijing, China). The hematoxylin-eosin (HE) staining kit was bought through Beyotime Biotechnology (Shanghai, China). Enzyme-linked immunosorbent assay (ELISA) kits for malondialdehyde (MDA), reactive oxygen species (ROS), testosterone (T), luteinizing hormone (LH) and follicle-stimulating hormone (FSH), were obtained from Herbal Source Biotechnology (Nanjing, China). AceQ^®^ Universal SYBR^®^ qPCR Master Mix was commercially procured from Vazyme Biotech (Nanjing, China). Real-time PCR plates (BBI, Shanghai, China; F603102-0001). Antibodies against S100A9, TLR4, NF-κB, p-NF-κB, IL-6, and TNF-α were procured from Proteintech (Wuhan, China). While for normalization control, β-actin antibody came from Cell Signaling Technology (Massachusetts, USA). In addition, Beyotime Biotechnology (Shanghai, China) supplied Actin-Tracker Green-488, Alexa Fluor 488-conjugated goat anti-rabbit IgG (H + L), and Alexa Fluor 555-labeled donkey anti-mouse IgG (H + L). Nuclear counterstaining mounting medium containing DAPI was provided by Biosharp Life Sciences (Anhui, China).

### Animals

Forty-two Male ICR mice at 8 weeks postnatal age (body weight 39.0 ± 2.0 g) were sourced from the Nantong University’s Experimental Animal Center. The subjects were maintained in an environment that met GLP standards, with temperatures ranging from 20 to 24 °C, humidity levels between 40 and 50%, and a daily cycle of 12 h of light followed by 12 h of darkness. Enough food and water were given for the voluntary feeding.

### Animal study approval

The study received clearance from the Animal Care and Use Committee at Nantong University and the Jiangsu Province Animal Care Ethics Committee. The approval document title is “The Effect and Mechanism of Tyrosol in Mice with Oligozoospermia” and the IACUC number is S20250420-009.

### Experimental treatments and sample collections

BUS was dissolved in a solution containing 10% DMSO in double distilled water, while tyrosol and hydroxytyrosol were dissolved in a solution comprising 2% DMSO and 98% corn oil. Mice were randomly assigned to six groups, with seven animals per group. Except for the control group, all mice received intraperitoneal injections of BUS 30 mg/kg b.w. to induce an oligozoospermia model (Wang et al., 2025; Ye et al., 2025; Wang et al., 2024a). Two weeks later, each group was treated as follows: (1) Control group: mice were provided with essential food and water; (2) BUS group (model group): mice received 200 µL of a mixture of 2% DMSO and 98% corn oil orally each day for 28 days; (3) 50 mg/kg hydroxytyrosol group: mice received 50 mg/kg hydroxytyrosol each day for 28 days, orally (Ye et al., 2024); (4) 10 mg/kg tyrosol group: mice received 10 mg/kg tyrosol orally each day for 28 days; (5) 30 mg/kg tyrosol group: mice received 30 mg/kg tyrosol each day for 28 days, orally; and (6) 50 mg/kg tyrosol group: mice received 50 mg/kg tyrosol orally each day for 28 days.

Following the final treatment, the mice were rendered unconscious by an intraperitoneal injection of 1% sodium pentobarbital at a dose of 50 mg/kg. Blood samples were then obtained *via* retro-orbital sinus puncture, followed by centrifugation at 3,000 g for 10 min at 4 °C. The serum was transferred into a new tube and kept at −80 °C for later analysis. All mice were weighed and euthanized *via* cervical dislocation, after which the testes and epididymides were excised and weighed individually. In each group, four testes from each group were fixed in 4% paraformaldehyde solution, while the remaining samples were rapidly frozen in liquid nitrogen and stored at −80 °C for immediate experimentation.

### Computer-assisted sperm analysis (CASA)

The two mL Tyrode’s solution was placed in a small dish with 37 °C-water bath. After mice euthanasia, the cauda epididymis was promptly excised from the mice and placed in a small dish. The tissue was then dissected to facilitate sperm release. The semen was incubated at 37 °C for 15 min and gently shaken to ensure even sperm distribution. Sperm concentration and motility were assessed using a computer-assisted sperm analysis system, Hamilton Thorne CEROS II, designed for both human and animal use.

### Periodic acid Schiff staining

Sperm was collected from the mouse epididymis and transferred onto slides, which were stored at −20 °C for furture experiments. After being left at room temperature for warming up approximately 20 min before being fixed in 4% paraformaldehyde for 15 min. The slides were then immersed in distilled water and stained using a periodic acid Schiff (PAS) Stain Kit (Beijing, Solibio, G11281) according to the manufacturer’s instructions. The slides underwent gradual dehydration in ethanol (70%, 80%, 95%, and 100%), followed by hyalinizing with xylene and sealing with neutral resin after staining.

### Histology and JOHNSEN score

After tissue collection, the testes and epididymis were immediately fixed in 4% paraformaldehyde for 24 h, followed by a 3–5 min rinse with running water. The specimens were then dehydrated with different concentrations of sucrose gradient solution, which included 10%, 20%, and 30%, with tissue sedimentation serving as the indicator for each dehydration step. 10-µm cryo-sections of tissue sections were cut by cryostat (Leica CM1950) and preserved at −20 °C for further experiment. Hematoxylin and eosin (Biosharp, Anhui, China) staining was performed according to the manufacturer’s protocol. Finally, the slices were observed and photographed by the Leica DM2500 microscope (Shanghai, China). Internationally, the commonly used JOHNSEN score is employed to evaluate testicular spermatogenic function. The score ranges from high to low, reflecting the degree of spermatogenic status within the testicles. Under the microscope, ten randomly selected seminiferous tubule fields of view are obtained. Each tubule is assigned a value according to the histological criteria of the JOHNSEN scoring table (10 points: normal spermatogenic function; 9 points: mild alteration in spermatogenic function, with numerous late-stage spermatids arranged in a disorderly manner and exfoliated spermatogenic cells within the lumen; 8 points: fewer than five spermatozoa per tubule, with few late-stage spermatids; 7 points: absence of spermatozoa and late-stage spermatids, but abundant early-stage spermatids; 6 points: absence of spermatozoa and late-stage spermatids, with few early-stage spermatids; 5 points: absence of spermatozoa and spermatids, but numerous spermatocytes; 4 points: absence of spermatozoa and spermatids, with few spermatocytes; 3 points: only spermatogonia present; 2 points: absence of spermatogenic cells, with only Sertoli cells present; 1 point: seminiferous tubules devoid of spermatogenic epithelium). The specific scores for each group are then calculated based on these assigned values.

### Western blot

The testis samples were homogenized using a 4 °C magnetic grinding instrument in radioimmunoprecipitation assay (RIPA) buffer until no visible tissue mass remained. The homogenate was maintained on ice for 20 min before being centrifuged at 12,000 g for an equal duration. The resulting supernatant, containing the protein fraction, was subsequently separated. Protein concentrations were determined using a BCA assay kit in a plate reader. Proteins, after being mixed with a suitable loading buffer, were denatured at 95 °C for 10 min and then separated by 10% or 12.5% SDS-PAGE. The proteins were transferred onto 0.22-µm PVDF membranes, which had been activated with methanol. Following electrophoresis and transfer, the membranes were blocked at room temperature with 5% skimmed milk for one hour, incubated overnight with the primary antibody at 4 °C, and finally exposed to the secondary antibody for one hour at room temperature.

### Immunofluorescence

The slides were removed from −20 °C storage and dried in a 37 °C oven for 30 min to prevent tissue detachment. The slides were rinsed 3 times with 1x PBS containing 1% Tween-20 for 5 min each, followed by one rinse in 1x PBS for 5 min. Tissue permeabilization was achieved by incubating the sample at room temperature for 30 min in a 0.3% Triton X-100 solution (Solarbio, Beijing, China). To block nonspecific binding, 3% donkey serum albumin was then applied for 1 h. Subsequently, the slides underwent an overnight incubation at 4 °C with the primary antibody against S100A9 (26992-1-AP, Proteintech, Wuhan, China; dilution 1:100). After rinsing with 1 × PBS, the sections were incubated with appropriately diluted secondary antibodies, Alexa Fluor 555-labeled IgG (A0453; Beyotime, 1:500), at room temperature for 1 h, followed by three additional 5-minute washes with PBS. Finally, the sections were mounted using a mounting medium containing DAPI (BL739B; Biosharp, Anhui, China) for nuclear counterstaining. Imaging was conducted with a confocal fluorescence microscope (TCSSP8; Leica).

### Enzyme-linked immunosorbent assay for T, FSH, LH, IL-1β, IL-6 and TNF-α

The serum was removed from −80 °C storage and put on ice to thaw gradually. Levels of Testosterone (T), follicle-stimulating hormone (FSH), luteinizing hormone (LH), IL-1β, IL-6, and TNF-α were measured using enzyme-linked immunosorbent assay (ELISA) kits from Herbal Source Biotechnology, Nanjing, China, following the manufacturer’s instructions. The minimum detectable concentrations for T, FSH, LH were 0.0375 nmol/L, 0.125 U/L, 17.5 pg/mL, respectively. Intra- and inter-assay coefficients of variation were both maintained below 10% for all assays.

### Enzyme linked immunosorbent assay for *alondialdehydem*, reactive oxygen species and superoxide dismutase

Testicular tissue is removed from −80 °C and weighed and then minced using small scissors before being placed in a grinding tube. The magnetic grinder was pre-cooled to 4 °C, and pre-cooled PBS was added to the grinding tube at a 1:9 ratio. The tube was placed on the grinder set to 60 Hz and processed until no visible tissue fragments remained. After centrifugation at 5,000 g for 10 min at 4 °C, the tissue suspension was carefully transferred to a flesh tube. Protein concentrations were quantified using a BCA Protein Assay Kit in a plate reader. Malondialdehyde (MDA), reactive oxygen species (ROS), and superoxide dismutase (SOD) levels were quantified using enzyme-linked immunosorbent assay kits (cby23821, cby18879, cby23823) from Herbal Source Biotechnology, Nanjing, China, following the manufacturer’s protocols. The assay sensitivities were 5 IU/mL for ROS, 0.075 nmol/L for MDA, and 0.78 IU/mL for SOD, with intra-assay and inter-assay coefficients of variation under 10%.

### RNA sequencing and data analysis

Mouse testes were processed to isolate total RNA using Trizol reagent (Vazyme). The testes were placed in new Rnase-free grinding tubes containing magnetic beads, after which one mL of Trizol was added. The mixture was ground at 70 Hz using a magnetic grinder at 4 °C until no visible tissue remained. Next, 200 µL of trichloromethane was added to the tube, followed by 15 s of vigorous shaking, and the mixture was allowed to equilibrate at ambient temperature for 5 min before being centrifuged at 10,000 ×g for 15 min at 4 °C. The aqueous phase was transferred to a flesh 1.5-mL Rnase-free tube, and total RNA was precipitated using isopropanol and washed with 75% ethanol. RNA quality and concentration were measured using a NanoDrop spectrophotometer (Thermo Fisher Scientific), while integrity was confirmed with a 2100 Bioanalyzer (Agilent Technologies, San Diego, CA, USA). The resulting RNA was subsequently employed to synthesize cDNA libraries for sequencing on the BGISEQ-500/MGISEQ-2000 System (BGI Shenzhen, China). Differential gene expressions were then analyzed, with functional annotations performed for Gene Ontology (GO) terms and Kyoto Encyclopedia of Genes and Genomes (KEGG) pathways using the clusterProfiler R package.

### Quantitative real-time PCR assays

Total RNA was extracted from mouse testes using Trizol reagent (R411; Vazyme, Nanjing, China), and RT-qPCR was utilized to evaluate target gene expression. RNA concentrations and integrity were verified before cDNA synthesis using an RNA reverse transcription kit. Concentrations of cDNA were quantified using a NanoDrop 2000 spectrophotometer, employing the GAPDH gene as an internal reference. Real-time quantitative PCR was performed in triplicate using ChamQ Universal SYBR qPCR Master Mix (Q711; Vazyme, Nanjing, China) on a CFX Connect Real-Time PCR Detection System (Bio-Rad). For detailed steps, please refer to the reagent manual. Primer sequences for RT-qPCR are provided in Table S1, and mRNA expression changes were calculated using the 2^−ΔΔCt^ method. The statistical power of this experimental design, calculated in RNASeqPower is 0.85.

### Statistical analysis

Data are expressed as mean ± SEM and analyzed with GraphPad Prism 8.0. Differences between two experimental groups were evaluated using Students’ t-tests, and one-way ANOVA was utilized for comparisons among multiple groups. Statistical significance was defined as a *P*-value of less than 0.05.

## Results

### Tyrosol (Tyr) alleviated testicular damage in busulfan-induced oligozoospermia mice

**Figure 1:**
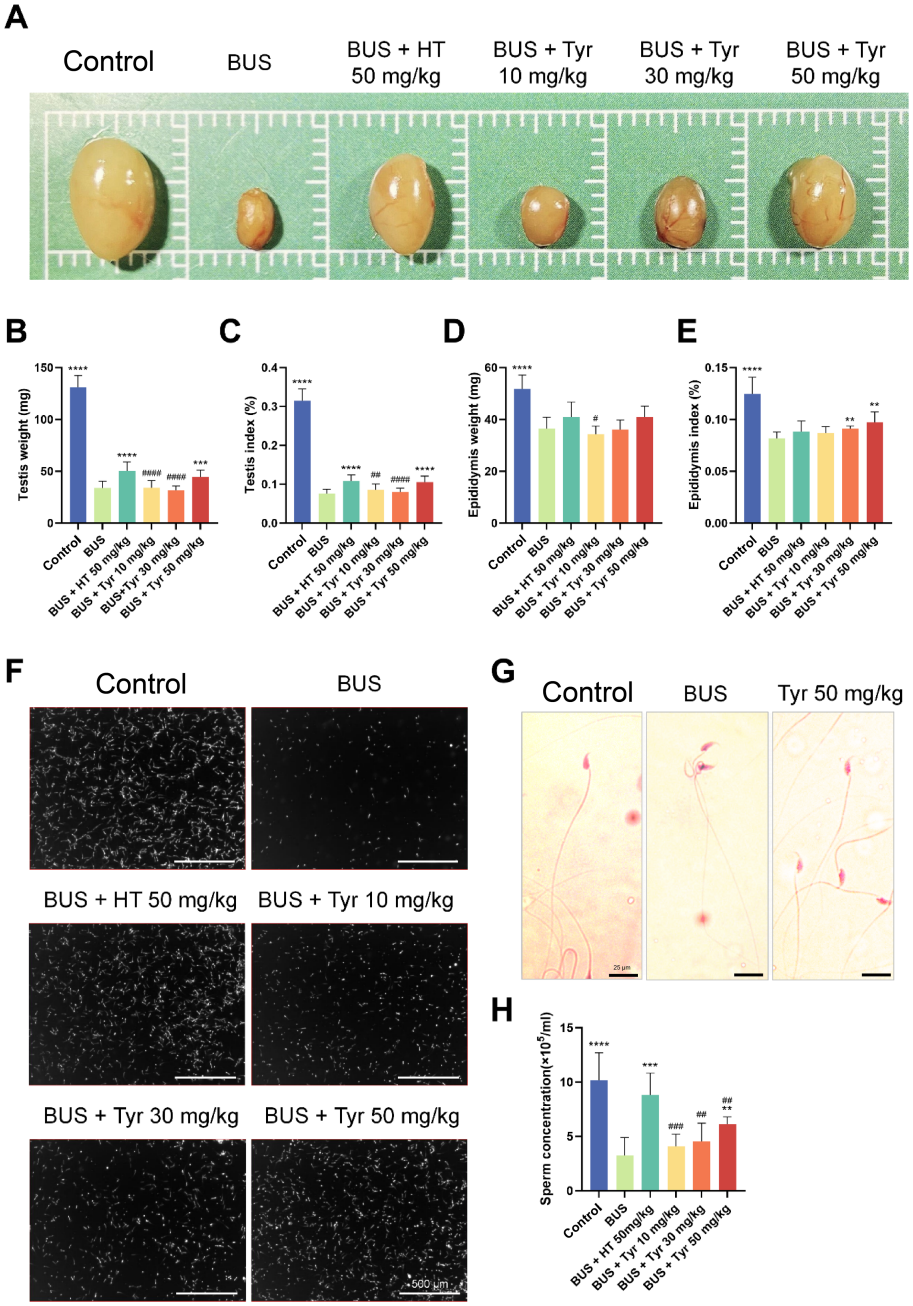
Tyrosol effects testis index, sperm concentration and sperm components. (A) Representative gross morphological images of the testes from 8-week-old mice after treatment with saline, BUS 30 mg/kg, BUS + HT 50 mg/kg, BUS + Tyr 10 mg/kg, BUS + Tyr 30 mg/kg, BUS + Tyr 50 mg/kg, (B) testicular weight, (C) testis ratio, (D) epididymis weight, (E) epididymis ratio, (F) epididymal sperm morphology, (G) acid-Schiff (PAS) staining of sperm, (H) Sperm concentration. Compared with: BUS group: *P < 0.05, **P < 0.01, ***P < 0.001, ****P < 0.0001. Compared with BUS + 50 mg/kg HT group: # *P* < 0.05, ## *P* < 0.01, ### *P* < 0.001, #### *P* < 0.0001. One-way analysis of variance (ANOVA) for analysis. Each column represents the mean ± SEM, *n* ≥ 7.

To investigate Tyr’s influenceon oligozoospermia development, we developed an oligozoospermia mouse model using busulfan and administered Tyr and hydroxytyrosol (3,4-dihydroxyphenylethanol, HT) to the mice (Fig. S1). Testis weight, testis index (testis weight/body weight * 100%), sperm concentration and sperm components show the construction of the oligozoospermia mouse model (Figs. 1A–1I). After 4 weeks of treatment, we evaluated testes size and observed that mice in the 50 mg/kg HT group and 50 mg/kg Tyr group exhibited larger testes compared to the model group (Fig. 1A). To obtain more information about this phenotype, testis weight and epididymis weight was measured, and we found that the 50 mg/kg HT group and 50 mg/kg Tyr group mice had dramatically increased testis weight and testis index compared to the model group, whereas no significant differences were observed in 10 mg/kg and 30 mg/kg Tyr group mice (Figs. 1B–1C). Additionally, treatment with both 30 mg/kg and 50 mg/kg Tyr improved the epididymal index compared to model group (Figs. 1D–1E). CASA demonstrated that administration of both 50 mg/kg Tyr and HT significantly increased sperm concentration but did not yield statistically significant improvements in sperm motility (Supplemental Material), suggesting that Tyr and HT partially reversed busulfan-induced oligozoospermia in mice (Figs. 1F, 1H). PAS glucogen staining of sperm showed that Tyr could recover the sperm abnormality induced by BUS (Fig. 1G).

### Tyr restores the morphological integrity of the testis and epididymis in oligozoospermia mice

**Figure 2:**
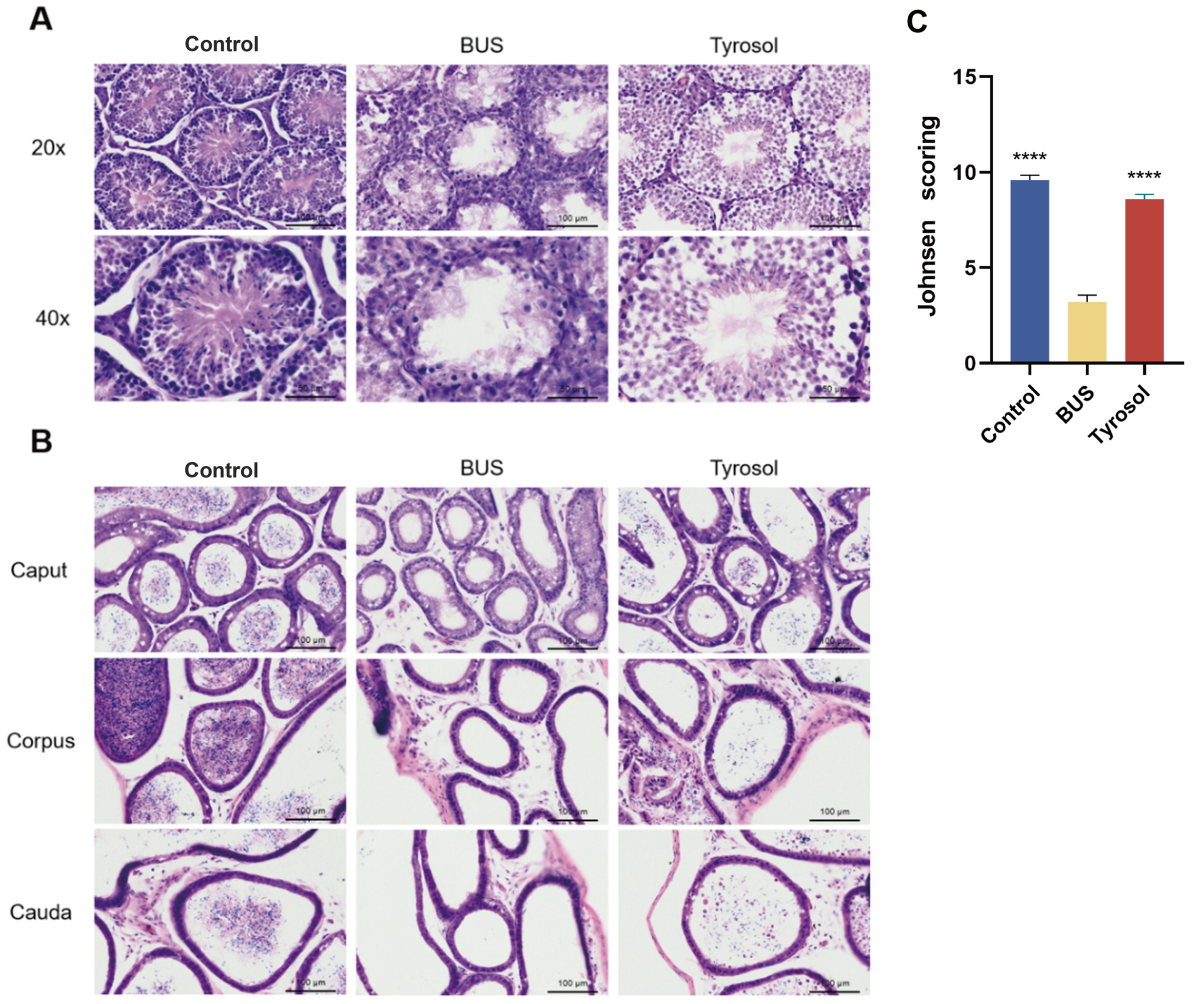
Histological analysis of mice testes and epididymis. (A) Morphology of testis. (B) Morphology of epididymis. Scale bar: 100 µm (the 20x), Scale bar: 50 µm (the 40x). (C) Johnsen scoring. The scores from 10 to 1 reflect a decline in testicular spermatogenic function from normal to severely impaired. Scores of 7 or below indicate the absence of spermatozoa. The JOHNSEN scoring table: 10 points: normal spermatogenic function; 9 points: mild alteration in spermatogenic function, with numerous late-stage spermatids arranged in a disorderly manner and exfoliated spermatogenic cells within the lumen; 8 points: fewer than five spermatozoa per tubule, with few late-stage spermatids; 7 points: absence of spermatozoa and late-stage spermatids, but abundant early-stage spermatids; 6 points: absence of spermatozoa and late-stage spermatids, with few early-stage spermatids; 5 points: absence of spermatozoa and spermatids, but numerous spermatocytes; 4 points: absence of spermatozoa and spermatids, with few spermatocytes; 3 points: only spermatogonia present; 2 points: absence of spermatogenic cells, with only Sertoli cells present; 1 point: seminiferous tubules devoid of spermatogenic epithelium. H&E staining for histological morphology observation. Compared with BUS group: ****P < 0.0001. Students’ *t*-tests for analysis. Each column represents the mean ± SEM, *n* ≥ 7.

To evaluate the effects of Tyr on the restoration of testicular and epididymal injuries in oligozoospermic mice, HE staining was performed to assess the morphology of the tissues in each group. Histological analysis revealed that in the model group, nearly all spermatogenic cells and mature spermatozoa within the seminiferous tubules of the testes were detached. Furthermore, a significant reduction in spermatogonia was observed, the Sertoli cells exhibited disorganized arrangements and the vacuolation was serious compared to the control group (Fig. 2A). After administering 50 mg/kg Tyr, we found that the vacuolation persisted, but the spermatogenic cells and mature spermatozoa were restored in the seminiferous tubules (Fig. 2A). In the epididymis, sperm were absent in the lumina of oligozoospermic mice, however, treatment with 50 mg/kg Tyr restored sperm levels. Mature spermatozoa and shed spermatogenic cells were observed in the epididymal lumina of the 50 mg/kg Tyr group (Fig. 2B). These findings indicate that 50 mg/kg Tyr administration improves the morphological integrity of the testes in oligozoospermic mice.

### Tyr improves the serum levels of T, FSH, LH in oligozoospermia mice

**Figure 3:**
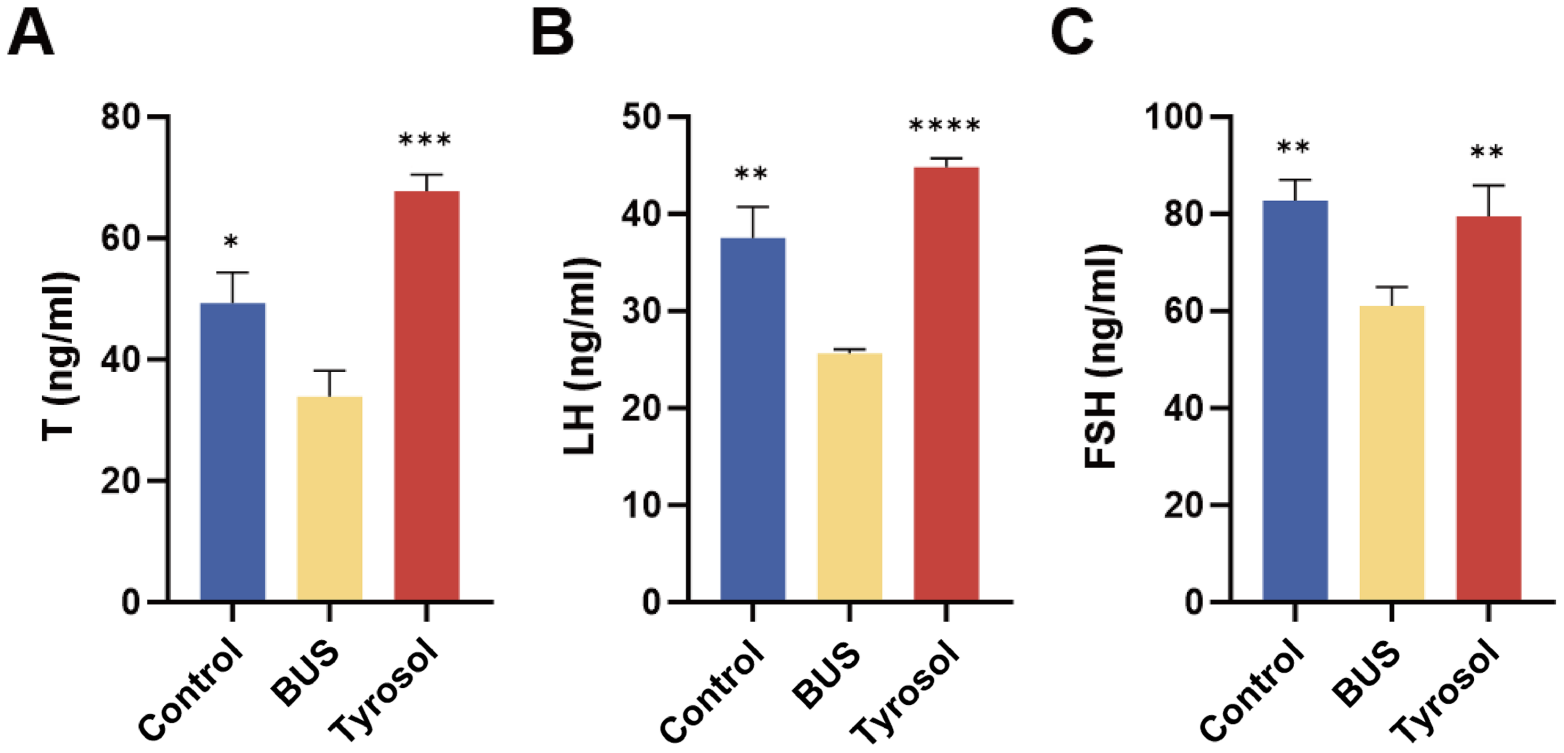
Effect of Tyr on hormone levels by ELISA. (A) T concentration. (B) LH concentration. (C) FSH concentration. Compared with BUS group: *P < 0.05, **P < 0.01, ***P < 0.001, ****P < 0.0001. Students’ t-tests for analysis. Each column represents the mean ± SEM, *n* ≥ 3.

Serum hormone levels, including testosterone (T), follicle-stimulating hormone (FSH), and luteinizing hormone (LH), are the essential indicators of gonadal function. In the model group, the levels of T, FSH, and LH were markedly reduced compared to those in the control group (*P* = 0.05, 0.01, 0.01, Figs. 3A–3C) However, administration of 50 mg/kg Tyr significantly improved serum hormone levels (*P* = 0.001, 0.0001, 0.01, Figs. 3A–3C), nearly approaching normal values. These findings suggest that Tyr enhances the serum levels of T, FSH, and LH in oligozoospermic mice.

### Tyr improves the serum levels of IL-1β, IL-6 and TNF-α in oligozoospermia mice

**Figure 4:**
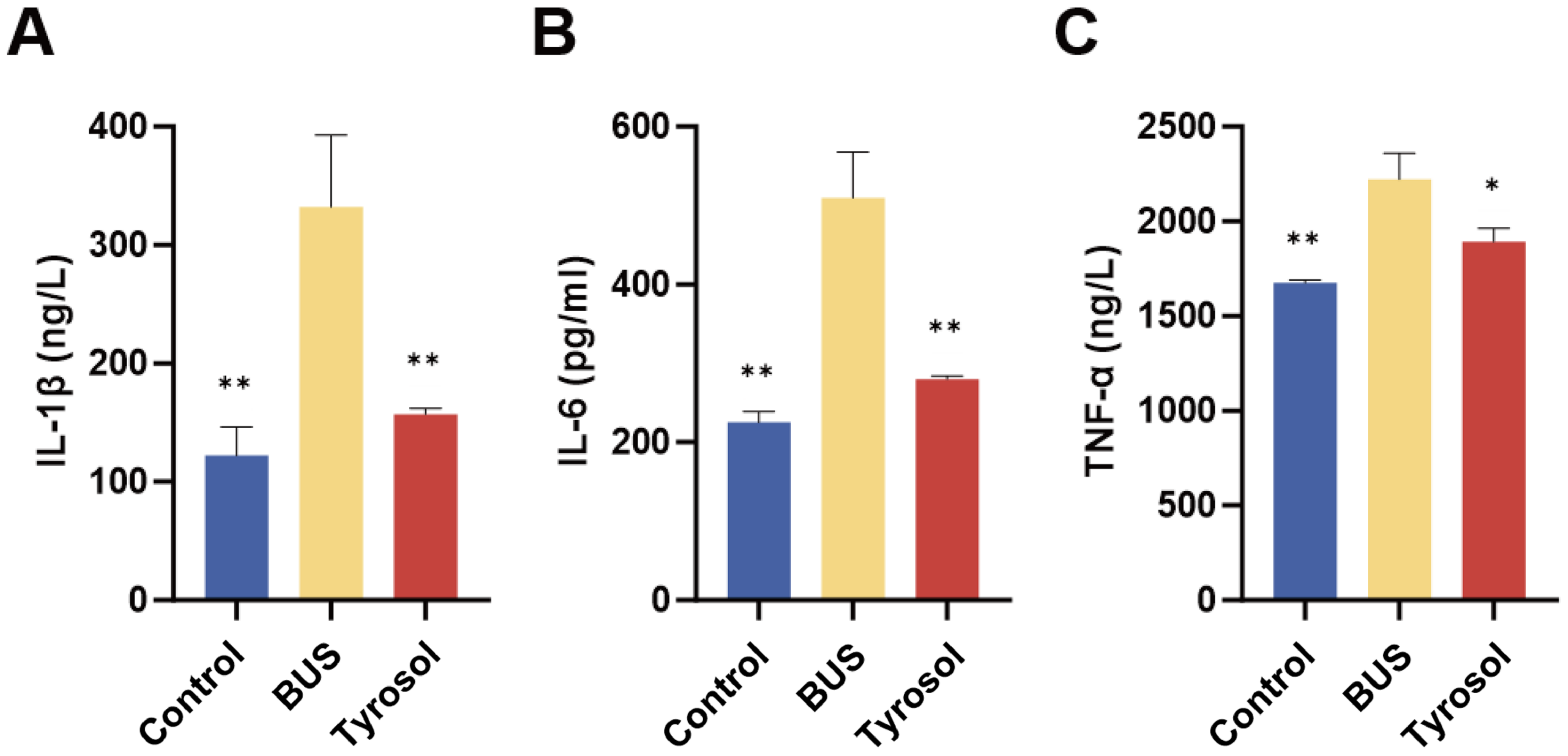
Effect of Tyr on inflammatory levels by ELISA. (A) IL-1β concentration. (B) IL-6 concentration. (C) TNF-α concentration. Compared with BUS group: *P < 0.05, **P < 0.01. Students’ *t*-tests for analysis. Each column represents the mean ± SEM, *n* ≥ 3.

Testicular inflammation is a major contributor to male infertility. Therefore, we examined the serum levels of inflammatory cytokines in mice. ELISA results indicated that the serum levels of IL-1β, IL-6, and TNF-α were markedly elevated in the model group compared to the control group (*P* = 0.01 for each, Figs. 4A–4C). Conversely, administering 50 mg/kg Tyr notably reduced cytokine levels, with IL-1β and IL-6 showing a significant decrease (*P* = 0.01, Figs. 4A–4B) and TNF-α exhibiting a reduction (*P* = 0.05, Fig. 4C). These findings suggest that Tyr effectively lowers serum inflammatory cytokine levels in oligozoospermic mice.

### Tyr improved oxidative indices in oligozoospermia mice

**Figure 5:**
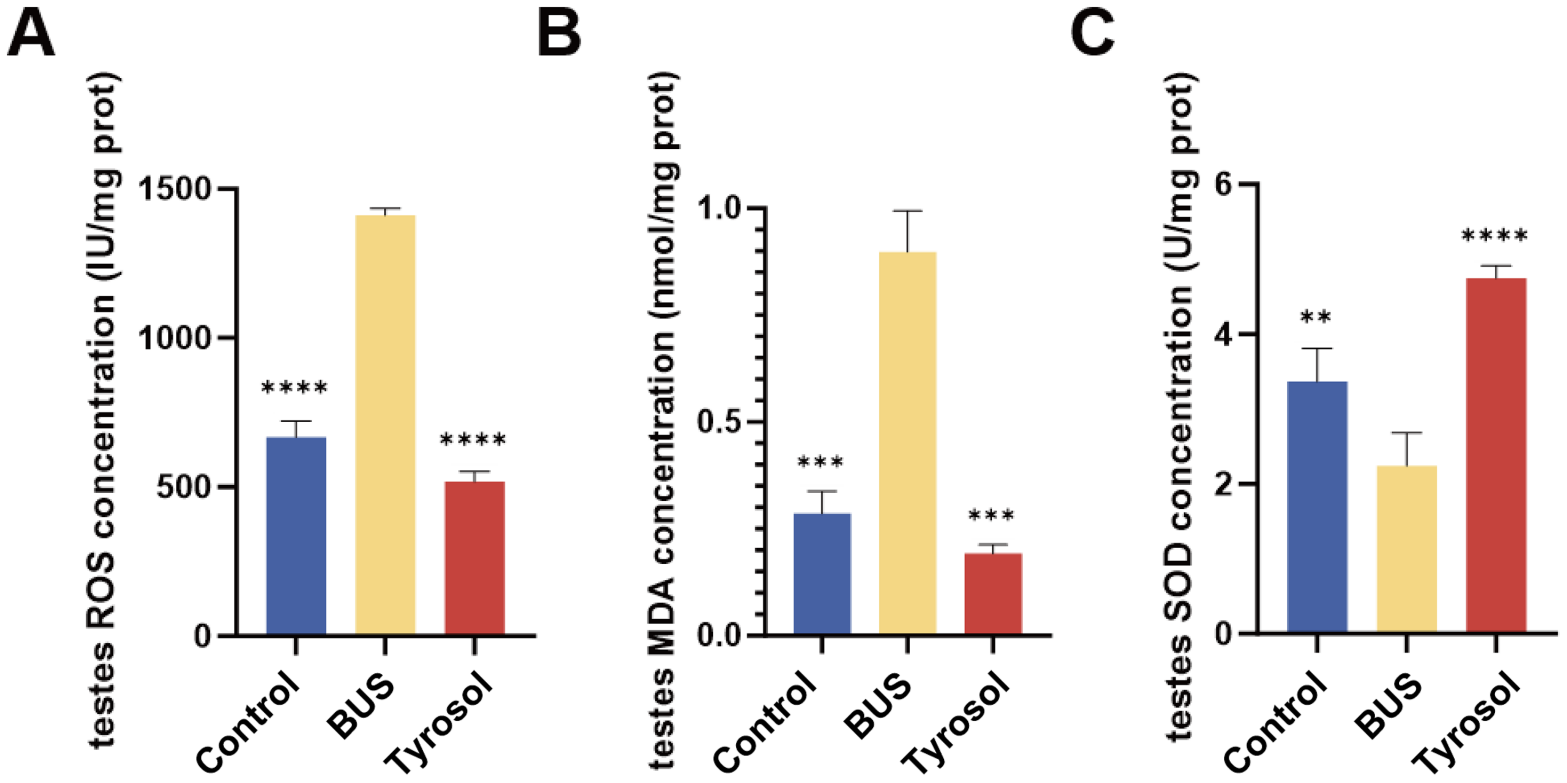
Effects of Tyr on oxidative indices by ELISA. (A) ROS concentration. (B) MDA concentration. (C) SOD concentration. Compared with BUS group: **P < 0.01, ***P < 0.001, ****P < 0.0001. Students’ *t*-tests for analysis. Each column represents the mean ± SEM, *n* ≥ 3.

Oxidative stress is a common feature of oligozoospermia. The findings demonstrated that testicular tissues from the model group exhibited significantly elevated concentrations of ROS and MDA relative to those of the control group (*P* = 0.0001 and 0.001, respectively, Figs. 5A–5B). Moreover, the levels of SOD, which removes the harmful superoxide anion radical (O^2−^), were notably diminished (*P* = 0.01, Fig. 5C). These observations suggest that the antioxidant capacity in oligozoospermic mice was reduced, and oxidative stress occurred to a certain extent. However, treatment with 50 mg/kg of Tyr significantly decreased ROS and MDA levels in the testes (*P* = 0.0001, 0.001, Fig. 5), bringing them closer to normal levels observed in control mice. In contrast, the SOD level significantly increased relatived to the model group (*P* = 0.0001, Fig. 5). These findings demostrated that Tyr treatment alleviates oxidative stress and restores antioxidant ability in oligozoospermia mice.

### Transcriptome sequencing of differentially expressed genes in testis of BUS and Tyr group

**Figure 6:**
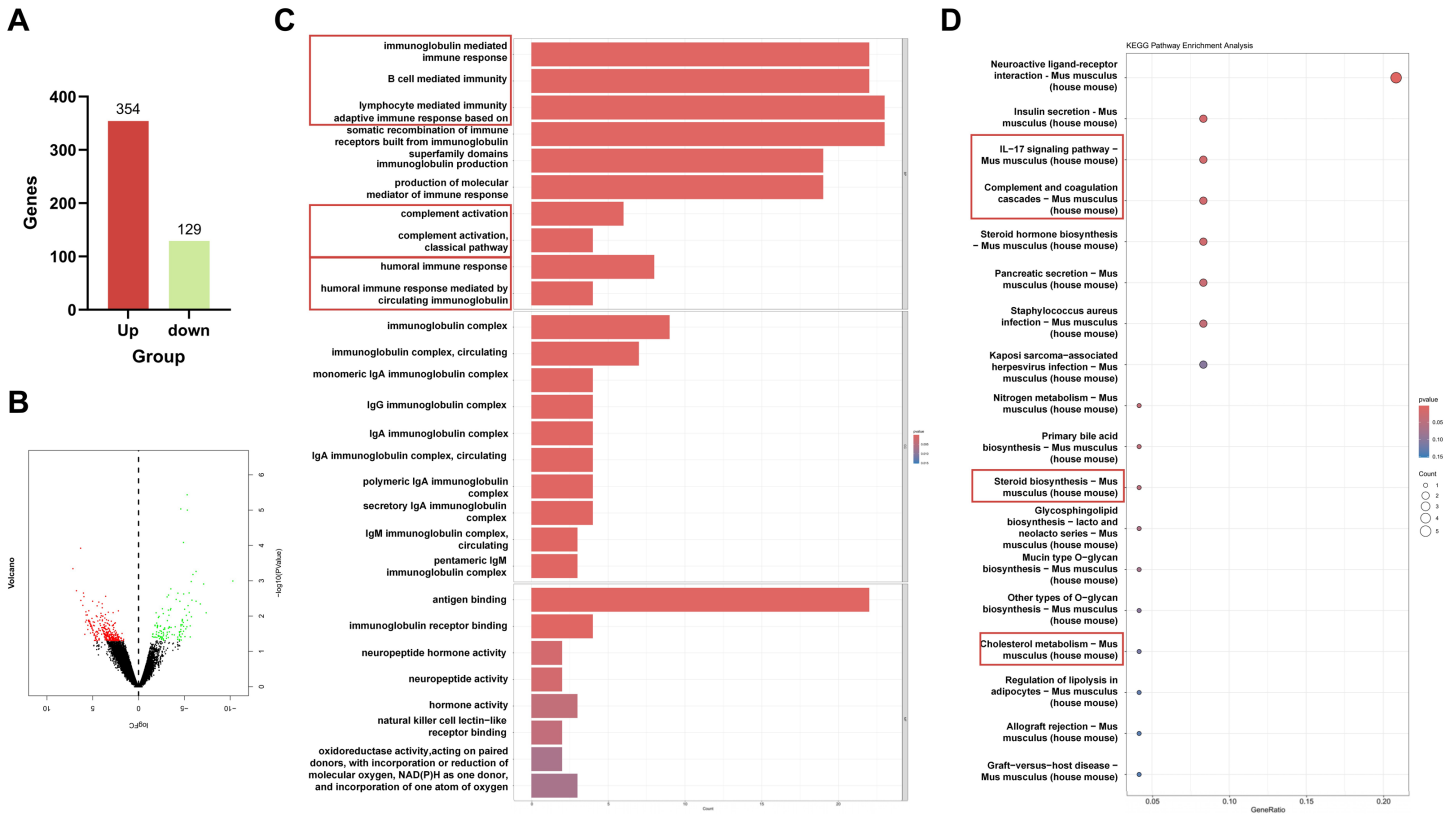
Analysis of RNA-seq. (A) Statistical evaluation of 354 upregulated and 129 downregulated genes. (B) Volcano plot for differentially expressed genes between the BUS group and the BUS+ Tyr 50 mg/kg group. (C) Gene Ontology (GO) enrichment analysis of upregulated genes in the BUS+ Tyr 50 mg/kg group. (D) KEGG pathways analysis for upregulated genes in the BUS+ Tyr 50 mg/kg group.

Based on the aforementioned findings, we inferred that the Tyr’s antioxidant and anti-inflammatory properties aid in treating oligozoospermia, and we hope to discover the regulatory genes by which Tyr improves the microenvironment for spermatogenesis in the mouse testis. Hence, we subsequently conducted transcriptome sequencing, GO analysis, and KEGG analysis on the testes of mice following 4 weeks of Tyr administration. By comparing the model group and the Tyr group, 354 up-regulated genes and 129 down-regulated genes were identified (Figs. 6A, 6B). Further GO classification analysis of the down-regulated differential genes revealed that the biological processes of genes related to “immunoglobulin mediated immune response” and “immune response” are involved in the 50 mg/kg Tyr treatment (Fig. 6C). Similarly, KEGG analysis of the downregulated genes identified enrichment in immune response pathways (Fig. 6D), among which the *S100A9* gene aroused our interest.

### Tyr modulates the S100A9/Tlr4/NF-κB pathway to improve testicular inflammation in oligozoospermic mice

**Figure 7:**
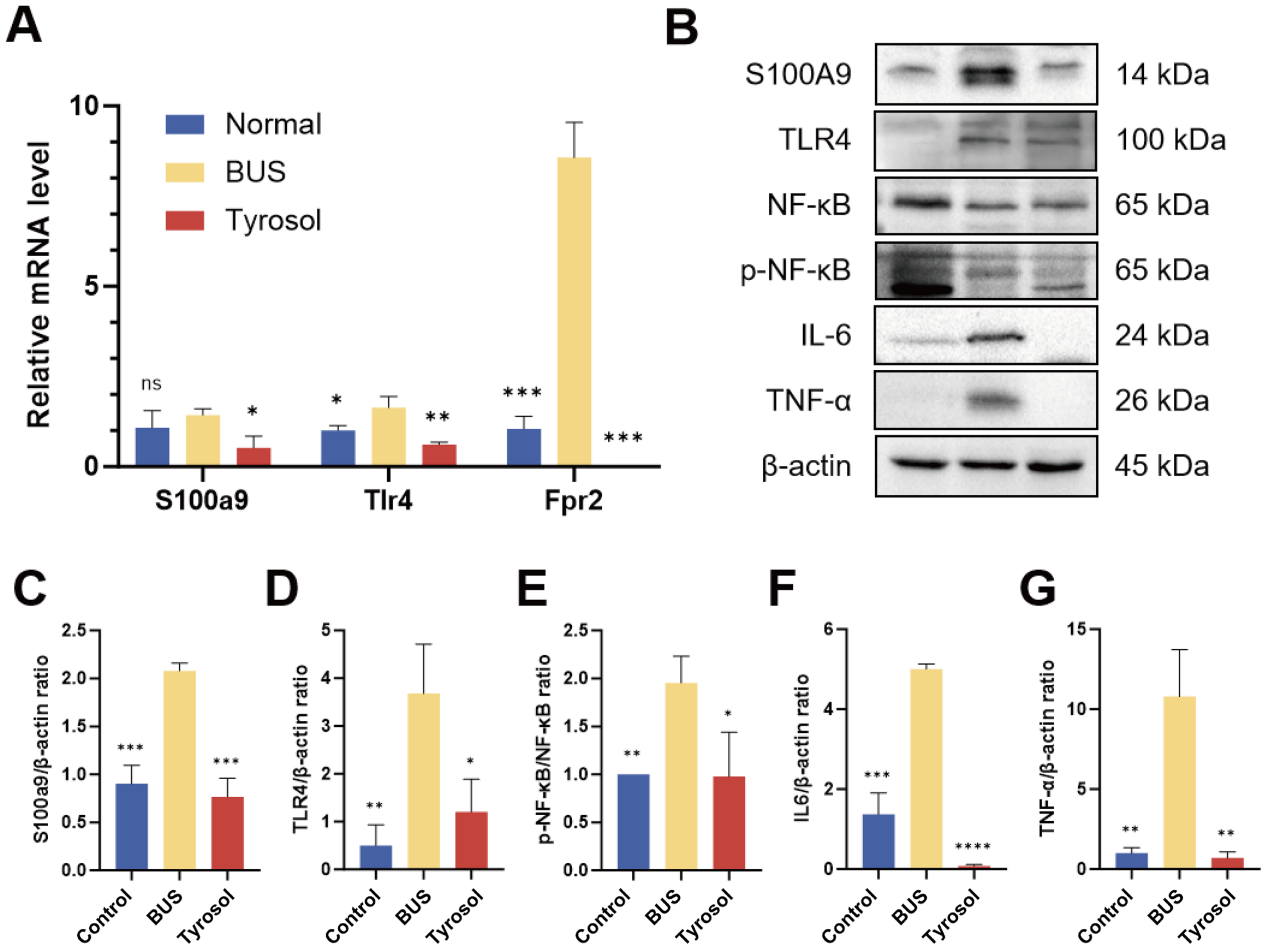
The effect of Tyr on inflammation induced by BUS. (A) Verification of sequencing results by RT-qPCR of S100A9, Tlr4 and Fpr2. (B–G) Western blot of relative proteins of inflammation. Compared with BUS group: *P < 0.05, **P < 0.01, ***P < 0.001, ****P < 0.0001. Students’ t-tests for analysis. Each column represents the mean ± SEM, *n* ≥ 3.

The RNA levels of S100A9 and its downstream targets, Tlr4 and Fpr2, were measured by RT-qPCR to validate the high-throughput sequencing results. Notably, the expressions of S100a9, Tlr4, and Fpr2 decreased following Tyr administration (*P* < 0.05 for S100a9, *P* < 0.01 for Tlr4, *P* < 0.001) (Fig. 7A). Furthermore, the expression of complement and congenital immune-related RNAs was lower in the Tyr-treated group relative to the BUS group (Fig. S2), consistent with the RNA sequencing results. We also assessed the protein levels of S100A9 and downstream regulators in mouse testicular tissue. S100A9 was sharply upregulated in oligozoospermia mice (*P* < 0.001), while Tyr administration notably reduced its levels (*P* < 0.001) (Figs. 7B, 7C). TLR4, a membrane protein receptor that binds to S100A9, showed increased expression in the BUS group (*P* < 0.01) and was restored after Tyr treatment (*P* < 0.05) (Figs. 7B, 7D). As a downstream molecule of TLR4, phosphorylated NF-κB mediates the nuclear translocation of p65/p50, promoting the production of inflammatory cytokines such as IL-1β, TNF-α, and IL-6. Western blot analysis revealed that the P-NF-κB/NF-κB ratio was notably elevated in the model group compared to the control group (*P* < 0.001), and Tyr treatment significantly improved this ratio (*P* < 0.0001) (Figs. 7B, 7E). Additionally, the levels of the inflammatory cytokines IL-6 and TNF-α were significantly increased in the testicular tissue of model group mice (*P* < 0.001, *P* < 0.01), while Tyr treatment significantly reduced these levels (*P* < 0.0001, *P* < 0.01) (Figs. 7B, 7F–7G). These findings suggest that Tyr treatment may mitigate testicular inflammation in oligozoospermia mice through the S100A9/Tlr4/NF-κB pathway.

### S100A9 expression was significantly decreased in testis of oligozoospermia mice

**Figure 8:**
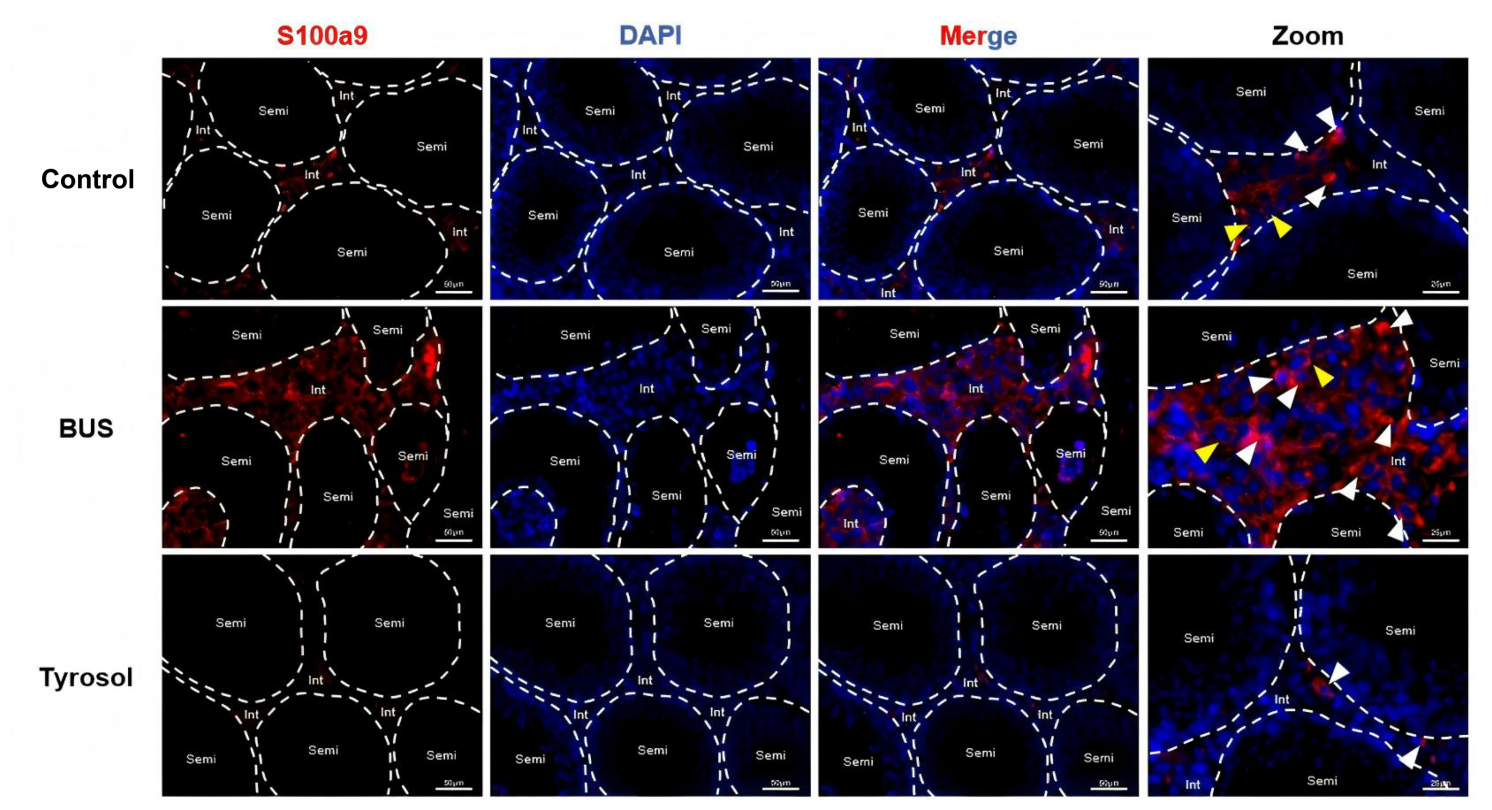
S100A9 expressed in testicular macrophages. Immunofluorescence staining analysis of S100A9 expression in testes, S100A9 (red), DAPI (blue), cytoplasm (white arrow) and the cell membrane (yellow arrow). Scare bar: 50 µm.

Immunofluorescence analysis was conducted to assess the expression and subcellular localization of S100A9 in the testes of different groups. A small amount of S100A9 was detected in the mouse testicular interstitium, where it localized to both the cytoplasm (white arrow) and the cell membrane (yellow arrow). Compared to the control group, testicular interstitial hyperplasia was increased, and S100A9 expression in the interstitium was significantly elevated in oligozoospermic mice. However, the S100A9 expression was markedly reduced following Tyr treatment, consistent with Western blot results. Furthermore, in the Tyr group, S100A9 was predominantly localized to cytoplasm (Fig. 8).

## Discussion

Tyrosol (Tyr) and hydroxytyrosol (HT) are olive-oil phenolics (Ditano-Vázquez et al., 2019; Kim et al., 2017; Adhami et al., 2015). Tyr, a white, water- and organic-soluble crystalline solid (López de las Hazas et al., 2015), is rapidly absorbed after oral intake, distributed *via* blood, and excreted as urinary glucuronides (Lee et al., 2016; Visioli et al., 2000). HT bears one extra hydroxyl group (Supplemental Material 1); this o-dihydroxyphenyl structure confers superior antioxidant potency (Karković Marković et al., 2019), whereas Tyr offers greater structural stability and resistance to auto-oxidation (Perona, Cabello-Moruno & Ruiz-Gutierrez, 2006). Both compounds display anti-inflammatory and antioxidant activities. HT is documented to cardiac protection (Poudyal et al., 2017; Frumuzachi et al., 2025), cancer prevention (Bernini et al., 2013; Hu et al., 2014), neuroprotection (Romanucci et al., 2020; D’Andrea et al., 2020; Yang et al., 2017), and the improvement of acute lung injury (Chen et al., 2025). Analogous benefits are emerging for Tyr, such as cardioprotective (Visioli et al., 2000), enhancing flap survival (Yu et al., 2024), improving ulcerative colitis (Yu et al., 2023), inhibiting diarrhea (Cellat et al., 2021) and alleviating pulmonary inflammation (Kargari et al., 2024).

In the field of male reproduction, numerous studies have suggested that HT can improve sperm quality. The addition of HT has been shown to enhance semem quality in various species, including chickens (Alharbi, Ali & Alharbi, 2024), horses (Sharafi et al., 2022), bulls (Arando Arbulu et al., 2021), deer (Baharsaadi et al., 2024), and humans (Kedechi et al., 2017). Co-incubation of HT with human sperm *in vitro* has demonstrated significant increases in sperm motility, reductions in sperm DNA oxidation, and lower ROS levels (Han et al., 2021; Alsemeh, Samak & El-Fatah, 2020). Additionally, HT has been shown to restore the histopathological structure of testicular tissue in diabetic and heat-stressed rats, improve sperm concentration and motility, and significantly reduce oxidative DNA damage and the apoptosis index (Sadeghirad et al., 2024; Neto et al., 2016). However, there is a lack of research on Tyr in the context of male reproduction. Our research demonstrated that Tyr possesses antioxidant properties and underlying mechanisms by which it mitigates BUS-induced oligozoospermia. Moreover, it has good safety and does not have hepatotoxicity or nephrotoxicity (Fig. S3), providing a foundation for its future development and clinical application.

In our research, treatment with 50 mg/kg of Tyr significantly improved testis weight, testicular index, and sperm concentration in oligozoospermic mice (Fig. 1). Additionally, Histological examination further revealed improvement of testicular morphology and a restoration of spermatogenesis within the seminiferous tubules following Tyr treatment (Fig. 2), a finding consistent with the research by Güvenç et al. (2020).

Spermatogenesis is a multifaceted process regulated by oxidative stress, the hormonal microenvironment, and intercellular interactions (Evans et al., 2021; Monceaux et al., 2022). Tyrosol has exhibited antioxidant activity in various tissues and organs, including the heart (Gabbia et al., 2023), kidneys (Zargari et al., 2023), and liver (Kutlu, Özkan & Güvenç, 2021; Kalaiselvan et al., 2016; You et al., 2025). Notably, studies have shown that tyrosol protects C57BL/6J mice retinal pigment epithelial cells from oxidative stress *via* the Nrf2/HO-1 pathway (Skorupskaite, George & Anderson, 2014). In our study, Tyr treatment reduced ROS and MDA levels in the testicular tissue of oligozoospermic mice compared to the BUS group, increased SOD levels, and effectively mitigated oxidative stress (Fig. 5). These findings are consistent with those reported by Güvenç et al. (2020). Nevertheless, our study did not further investigate the molecular mechanisms by which tyrosol modulates oxidative stress in the testes of oligozoospermic mice.

Testosterone, a steroid hormone secreted by Leydig cells, plays a crucial role in regulating spermatogenesis and maintaining testicular homeostasis. Its biosynthesis is tightly regulated by the hypothalamic-pituitary-gonadal (HPG) axis. Neurons in the hypothalamic arcuate nucleus release gonadotropin-releasing hormone (GnRH) in a pulsatile manner, activating anterior pituitary gonadotrophs, which in turn synthesize luteinizing hormone (LH) and follicle-stimulating hormone (FSH) (Yen, 1975; Kooshesh et al., 2020). Notably, oxidative stress can impair the functional integrity of the HPG axis by inducing degeneration of GnRH neurons, leading to reduced LH/FSH secretion and dysregulated testosterone production in the testis (Alkhateeb et al., 2019). Therefore, we assessed the serum levels of key hormones to investigate whether Tyr affects hormone homeostasis in oligozoospermic mice. The results showed that Tyr treatment improved serum T, FSH, and LH levels in oligozoospermic mice (Fig. 3). These findings suggest that Tyr may enhance spermatogenesis in oligozoospermic mice by restoring hormonal balance.

High-throughput transcriptomic sequencing was employed to investigate the underlying mechanisms (Fig. 6). Gene ontology analysis revealed that S100A9, a key component of the S100A9/TLR4/NF-κB pathway, was notably reduced in the testes of mice following Tyr administration relative to the model group, a finding corroborated by RT-qPCR results (Fig. 7). S100A9, a calcium-binding protein primarily derived from myeloid cells—including neutrophils, monocytes, and dendritic cells—plays pivotal roles in innate immune responses, macrophage activation, and inflammation-associated tissue damage (Silva de Melo et al., 2023; Vogl et al., 2018). During infection, S100A9 induces immune cell chemotaxis, macrophage activation, and the production of inflammatory factors to promote inflammation (Quoc et al., 2021). Moreover, S100A9 facilitates the differentiation of macrophages into the M1 phenotype *via* the TLR4/MyD88/NF-κB signaling axis, thereby recruiting neutrophils and stimulating the secretion of pro-inflammatory cytokines such as IL-1β and TNF-α, which intensifies tissue inflammation (Gong et al., 2024). Additionally, S100A9 promotes neutrophil extracellular trap formation through activation of the p38 MAPK and ERK pathways, further contributing to inflammation and type 2 neutrophilic inflammation (Husain & Somani, 1998). Inflammation-induced testicular oxidative stress is a common cause of male infertility (Shen et al., 2024). Numerous studies have confirmed that the S100A9/TLR4/NF-κB pathway enhances inflammatory responses. Within the male reproductive system, S100A9 serves as a potential inflammatory marker (Martínez-Heredia et al., 2008). In experimental autoimmune orchitis in rats, S100A9 levels in seminal plasma and testicular macrophages were significantly elevated, and increased S100A9 expression has also been observed in patients with asthenozoospermia compared to normal semen (Martínez-Heredia et al., 2008). Our findings indicate that, compared with the model group, Tyr administration sharply decreased the S100A9 and TLR4 levels in the testicular S100A9/TLR4/NF-κB pathway, as well as downstream NF-κB levels, resulting in reduced expression of IL-6 and TNF-α proteins (Fig. 7). Immunofluorescence analysis demonstrated that S100A9 was predominantly localized in the testicular interstitium of adult mice (Fig. 8), consistent with previous reports by Fan et al. (2021). Tyr and its metabolites may inhibit the S100A9/TLR4/NF-κB pathway by reducing S100A9 and TLR4 levels in the testis. This inhibition may suppress macrophage activation and the production of pro-inflammatory cytokines, thereby alleviating testicular inflammation. However, it remains unclear whether Tyr or a specific metabolite is primarily responsible for reducing S100A9 levels and which testicular cell types modulate the S100A9/TLR4/NF-κB pathway to inhibit inflammation.

## Conclusions

**Figure 9:**
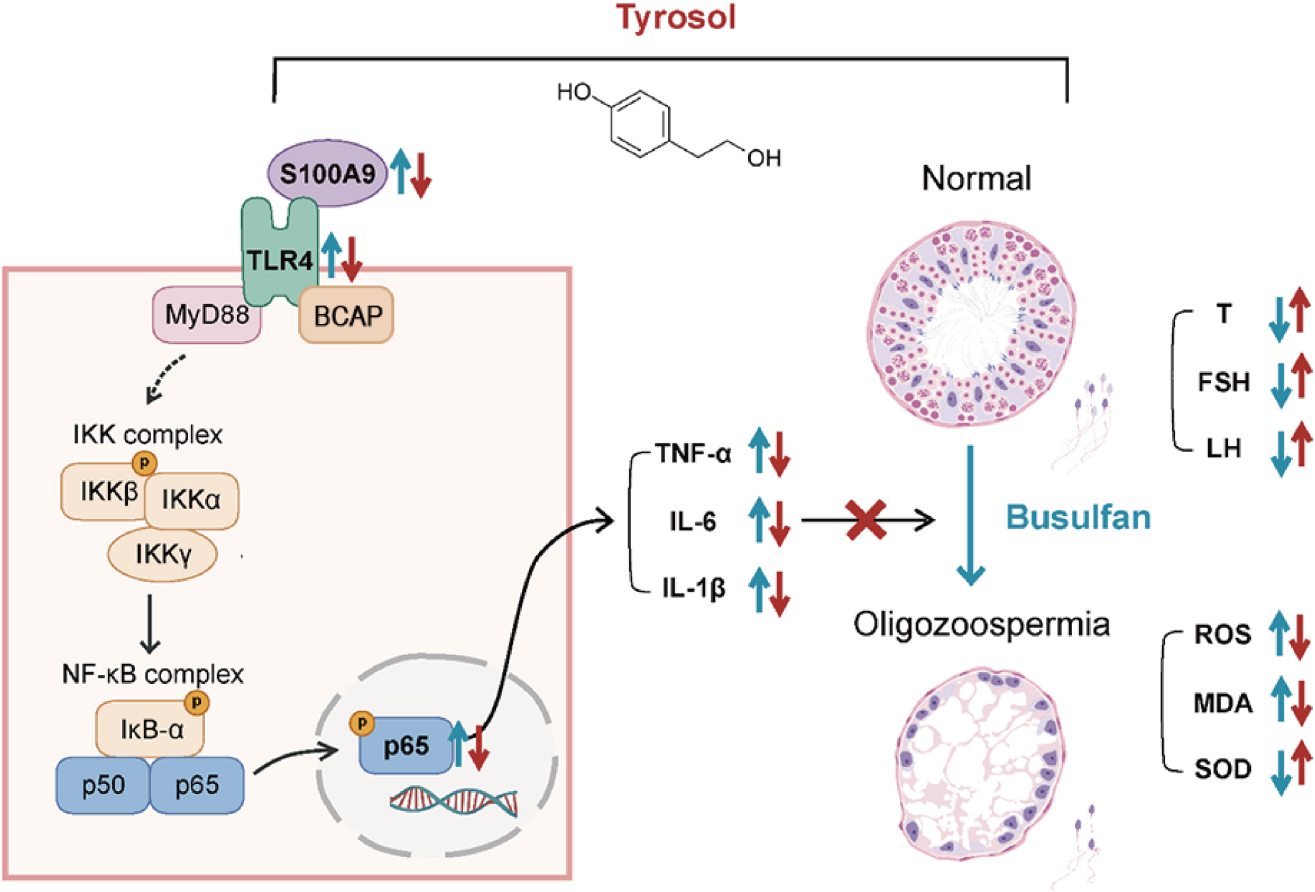
Proposed mechanism by which Tyr ameliorates oligozoospermia. BUS can lead to high oxidative levels in the testes, thereby enhancing the S100A9/TLR4/NF-κ B pathway, which exacerbates inflammatory responses and disrupts spermatogenesis; whereas Tyr can reduce the levels of S100A9 and TLR4 in the testes, inhibit macrophage activation and the production of pro-inflammatory cytokines, thereby alleviating testicular inflammation and restoring spermatogenesis.

Overall, our study elucidates a partial mechanism underlying inflammation in busulfan-induced oligozoospermic mice, implicating the S100A9/TLR4/NF-κB pathway in testicular inflammation. Administration of Tyr significantly reduces S100A9 and TLR4 expression in the testis, thereby ameliorating inflammation. Additionally, Tyr improves sex hormone balance, oxidative stress levels, and inflammatory status, ultimately enhancing sperm concentration and restoring testicular tissue architecture in oligozoospermia (Fig. 9). Future studies will focus on cellular-level investigations to elucidate the specific effects of Tyr on different testicular cells.

##  Supplemental Information

10.7717/peerj.20887/supp-1 Supplemental Information 1 Raw Data

10.7717/peerj.20887/supp-2 Supplemental Information 2 Supplemental figures and table

10.7717/peerj.20887/supp-3 Supplemental Information 3 MIQE checklist

10.7717/peerj.20887/supp-4 Supplemental Information 4 The ARRIVE guidelines 2.0 author checklist

## Additional Information and Declarations

### Data Availability

The following information was supplied regarding data availability: The data is available at Figshare: Wang, Weizhen (2025). Original data. figshare. Dataset. https://doi.org/10.6084/m9.figshare.28748561.v1
The RNA-Seq Data is available at Figshare: Wang, Weizhen (2026). Tyrosol effectively improves spermatogenesis recovery by S100A9/TLR4/NF-κ B pathway in busulfan-induced oligozoospermia mice. figshare. Dataset. https://doi.org/10.6084/m9.figshare.31077541.v1
The RNA-Sequence data is available at CNGB: https://doi.org/10.26036/CNP0008978
