# Supplementary material for: Tyrosol effectively improves spermatogenesis recovery by S100A9/TLR4/NF-κB pathway in busulfan-induced oligozoospermia mice
Source: PeerJ. 2026 Mar 30;14:e20887. doi: 10.7717/peerj.20887 (PMC13045846; doi:10.7717/peerj.20887)
Supplement: Supplemental Information 2 [file peerj-14-20887-s002.docx]

**Supplemental Information**

**Tyrosol effectively improves spermatogenesis recovery by** **S100A9/TLR4/NF-κB pathway in busulfan-induced** **oligozoospermia mice**

Weizhen Wang^1,2†^, Wenbiao Zhou^2†^, Xiaoran Li^2^, Taowen Ye^2^, Wanqing Zhu^2^, Guanning Zhong^2^, Chengniu Wang^2^, Haiying Geng^3^, Xiaofang Tan^1*^, Ruikun Hu^1*^, Xiaorong Wang^1*^

^1^ Center for Reproductive Medicine, Affiliated Maternity and Child Healthcare Hospital of Nantong University, Nantong, Jiangsu, China

^2^ Institute of Reproductive Medicine, Medical School, Nantong University, Nantong, Jiangsu, China

^3^ Department of Clinical Laboratory, Affiliated Maternity and Child Health Care Hospital of Nantong University, Nantong, Jiangsu, China

Corresponding Author:

Xiaorong Wang

Affiliated Maternity and Child Healthcare Hospital of Nantong University. 399 Century Avenue, Nantong, Jiangsu, 2260001, China

Email address: [xr0104@ntu.edu.cn](mailto:xr0104@ntu.edu.cn)

Ruikun Hu

Affiliated Maternity and Child Healthcare Hospital of Nantong University. 399 Century Avenue, Nantong, Jiangsu, 2260001, China

Email address: huruikun2021@163.com

Xiaofang Tan

Affiliated Maternity and Child Healthcare Hospital of Nantong University. 399 Century Avenue, Nantong, Jiangsu, 2260001, China

Email address: txf15@163.com


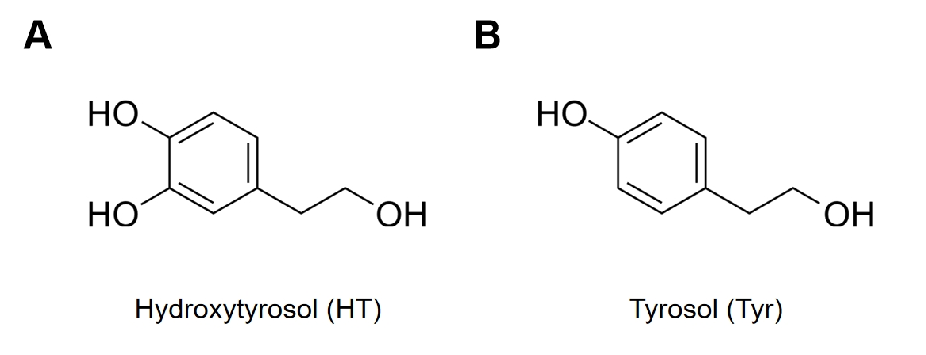


**Figure S1. Chemical structure of Tyrosol and Hydroxytyrosol.** (A): Hydroxytyrosol (HT), (B): Tyrosol (Tyr).


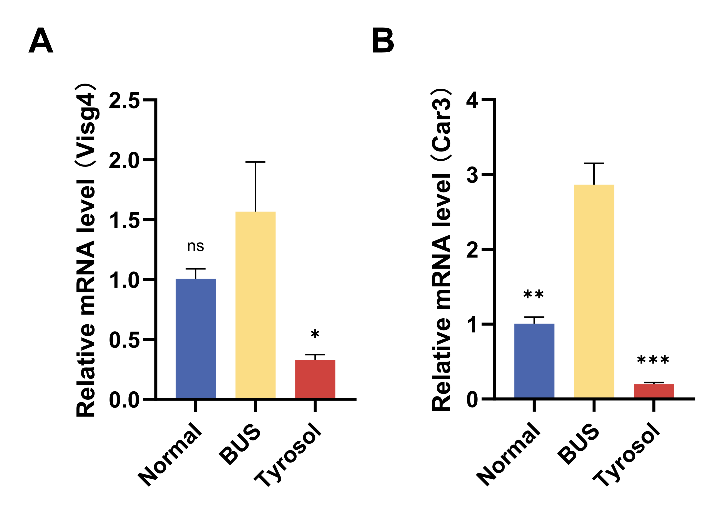


**Figure S2.** Verification and analysis of sequencing results by RT-qPCR. vs. BUS group: *P < 0.05, **P < 0.01, ***P < 0.001, n = 3.


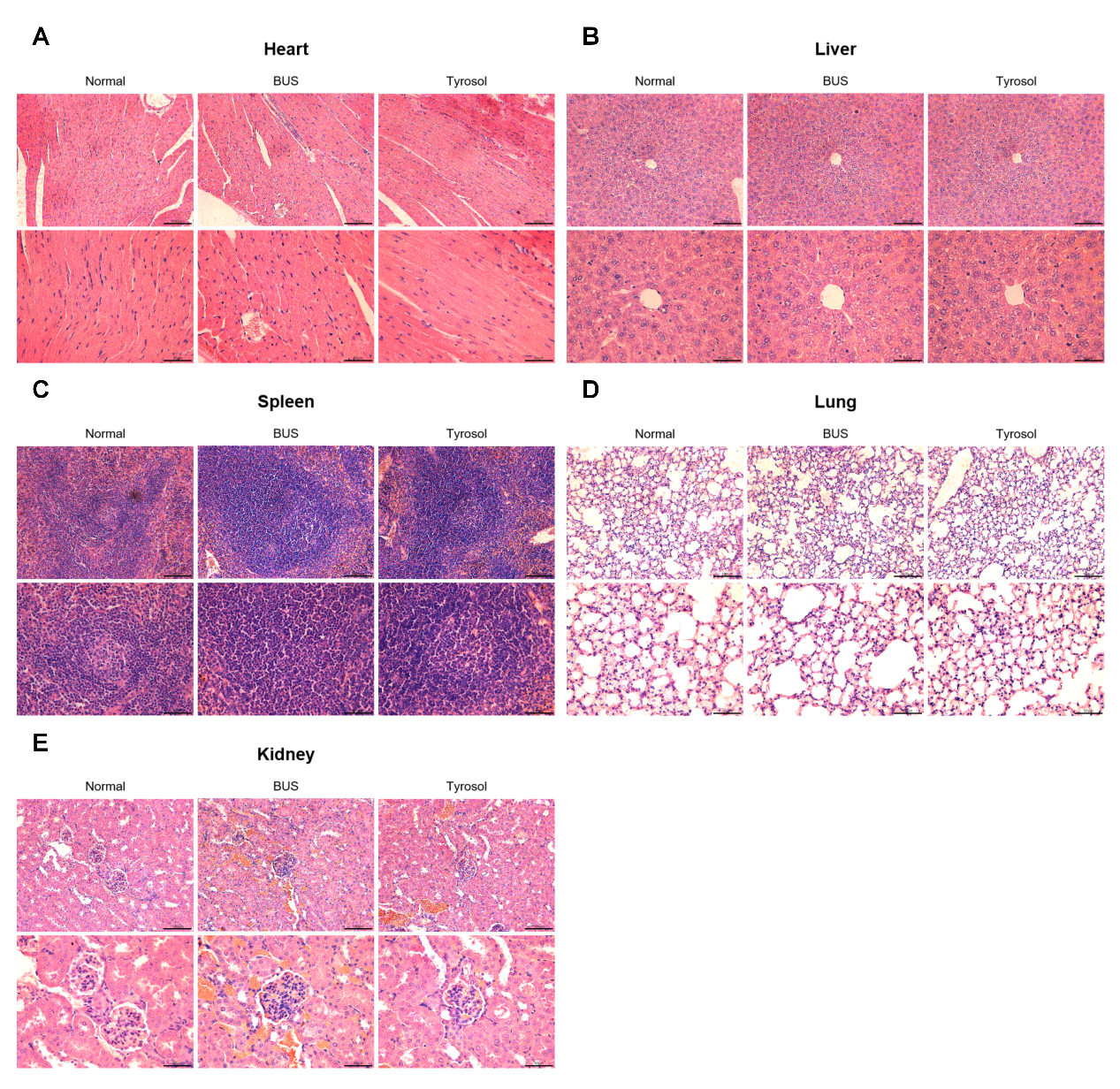


**Figure S3.** Histological examination of mice organs. (A): Morphology of heart. (B): Morphology of liver. (C): Morphology of spleen. (D): Morphology of lungs. (E): Morphology of kidney.

**Table S1**. Primer Sequences for Real-Time Quantitative PCR.

| Name | Primer Sequences | Accession Number | Primer Efficiency, % |
| --- | --- | --- | --- |
| Gapdh-F | AGGTCGGTGTGAACGGATTTG | NM_001289726.2 | 90 |
| Gapdh-R | GGGGTCGTTGATGGCAACA |  |  |
| S100a9-F | ATACTCTAGGAAGGAAGGACACC | NM_009114.3 | 94 |
| S100a9-R | TCCATGATGTCATTTATGAGGGC |  |  |
| Fpr2-F | GAGCCTGGCTAGGAAGGTG | NM_008039.2 | 90 |
| Fpr2-R | TGCTGAAACCAATAAGGAACCTG |  |  |
| Tlr4-F | AGGCACATGCTCTAGCACTAA | NM_021297.2 | 95 |
| Tlr4-R | AGGCTCCCCAGTTTAACTCTG |  |  |
| Vsig4-F | AGAGGCTACAGGCAAGTTTTG | NM_177789.5 | 87 |
| Vsig4-R | GGAGTCACGTAGGAAGATGGT |  |  |
| Car3-F | TGACAGGTCTATGCTGAGGGG | NM_007606.3 | 92 |
| Car3-R | CAGCGTATTTTACTCCGTCCAC |  |  |
